# Deep Artificial Neural Networks for the Diagnostic of Caries Using Socioeconomic and Nutritional Features as Determinants: Data from NHANES 2013–2014

**DOI:** 10.3390/bioengineering5020047

**Published:** 2018-06-18

**Authors:** Laura A. Zanella-Calzada, Carlos E. Galván-Tejada, Nubia M. Chávez-Lamas, Jesús Rivas-Gutierrez, Rafael Magallanes-Quintanar, Jose M. Celaya-Padilla, Jorge I. Galván-Tejada, Hamurabi Gamboa-Rosales

**Affiliations:** 1 Unidad Académica de Ingeniería Eléctrica, Universidad Autónoma de Zacatecas, Jardín Juarez 147, Centro, Zacatecas 98000, Zac, México; lzanellac@uaz.edu.mx (L.A.Z.-C.); tiquis@uaz.edu.mx (R.M.-Q.); gatejo@uaz.edu.mx (J.I.G.-T.); hamurabigr@uaz.edu.mx (H.G.-R.); 2 Unidad Académica de Odontología, Universidad Autónoma de Zacatecas, Jardín Juarez 147, Centro, Zacatecas 98000, Zac, México; nubiachavez@uaz.edu.mx (N.M.C.-L.); rigj002959@uaz.edu.mx (J.R.-G.); 3 CONACYT—Universidad Autónoma de Zacatecas—Jardín Juarez 147, Centro, Zacatecas 98000, Zac, Mexico; jose.celaya@uaz.edu.mx

**Keywords:** NHANES, oral health, dental caries, classification multivariate models, computer-aided diagnosis, artificial neural networks, deep learning, statistical analysis

## Abstract

Oral health represents an essential component in the quality of life of people, being a determinant factor in general health since it may affect the risk of suffering other conditions, such as chronic diseases. Oral diseases have become one of the main public health problems, where dental caries is the condition that most affects oral health worldwide, occurring in about 90% of the global population. This condition has been considered a challenge because of its high prevalence, besides being a chronic but preventable disease which can be caused depending on the consumption of certain nutritional elements interacting simultaneously with different factors, such as socioeconomic factors. Based on this problem, an analysis of a set of 189 dietary and demographic determinants is performed in this work, in order to find the relationship between these factors and the oral situation of a set of subjects. The oral situation refers to the presence and absence/restorations of caries. The methodology is performed constructing a dense artificial neural network (ANN), as a computer-aided diagnosis tool, looking for a generalized model that allows for classifying subjects. As validation, the classification model was evaluated through a statistical analysis based on a cross validation, calculating the accuracy, loss function, receiving operating characteristic (ROC) curve and area under the curve (AUC) parameters. The results obtained were statistically significant, obtaining an accuracy ≃ 0.69 and AUC values of 0.69 and 0.75. Based on these results, it is possible to conclude that the classification model developed through the deep ANN is able to classify subjects with absence of caries from subjects with presence or restorations with high accuracy, according to their demographic and dietary factors.

## 1. Introduction

Chronic diseases are the main problems in public health worldwide. The pattern of disease has been transformed and oral diseases are considered one of the main public health problems due to its high incidence and prevalence in all regions of the world, and, as in all diseases, the greatest burden is on populations disadvantaged and socially marginalized; treatment of the conditions is extremely expensive and is not feasible in most low and middle income countries. This characteristic represents an important problem, since, according to the World Health Organization (WHO), oral diseases are the fourth most expensive cause to treat in the most industrialized countries [1].

Oral health is an essential component for quality of life of people due to its influence as a determinant factor in general health of individuals and communities becoming a relevant point in health care. Therefore, the serious repercussions in terms of pain and suffering, impairment of function and effect on quality of life should also be considered. An important aspect to consider is that oral diseases increase the risk of chronic diseases, such as: cardiovascular and cerebrovascular, diabetes mellitus and respiratory. On the other hand, epidemiological surveillance of oral diseases becomes important insofar as it provides useful elements for the planning, programming, organization, integration, control and direction of the oral health program, at the same time that it guides the attention to the population [2].

Caries is the most frequent condition and according to the WHO; it affects between 60% and 90% of children of school age between 5 to 17 years old. The determinants and conditions of oral health status are multicausal, multisectoral and interdisciplinary categories that encompass a series of situations related to the historical and political process that each country experiences. In addition, the report of the Organización Panamericana de la Salud (OPS) describes how the development of this condition depends on the frequency of carbohydrate consumption, the characteristics of the food, the time exposure, plaque removal and susceptibility of the guest, adding the few preventive measures in oral health and the difficulty of making use of specialized dental medical services. These factors interact simultaneously, and their variables correspond to different orders from biological processes, to complex historical-cultural structures and social relationships, socioeconomic level, educational level, among others, making health phenomena complex [3,4].

There are too many risk factors and important determinants related to the presence of caries, and it is clear that the greater the degree of risk exposure, the greater the probability of contracting or developing it. Due to the difficulty of representing the control of the incidence of caries due to its high prevalence and the large number of factors that can influence this state, at present, some studies have implemented algorithms and performed analyses based on computer-aided diagnosis (CAD) where prediction models are used when it is necessary to know in the future the behavior of highly related complex data; these have utilities in clinical and research dentistry [5].

An example of these algorithms are the artificial neural networks (ANNs), a method used for the prediction of diseases, such is the case of oral diseases, as well as being mathematic models based on a principle of learning that is based on the concepts of artificial intelligence and the biological response of the human brain. Likewise, they are involved in systems’ construction processes that allow classifying, modeling and predicting information. ANNs are semiparametric nonlinear models, which allow the integration of variables and easily handle large amounts of data compared to linear analyses. They have processing elements or neurons, which are the units of the system that can be adjusted or trained through a process of learning and generalization. The information of each of these neurons is grouped and processed [6].

According to the literature, dental caries incidence has been a problem that has been studied by a series of researchers, trying to identify caries risk determinants. Lam et al. [7] found that dental visits, brushing frequency, lower parental perceived importance of baby teeth, and weaning onto solids are determinants associated with plaque accumulation, validating these results specifically in children. In the work of Fernandes et al. [8], a cross-sectional study of 274 children and their mothers based on demographic/socio-economic status is presented; through a Poisson regression approach, an analysis was performed, obtaining that dental caries can be mainly found in mothers of children aged 1, demonstrating the relationship between demographic/socio-economic status and caries.

As mentioned in the text, there are different types of risk factors that are associated with genetic and nutritional data. Lips et al. [9] presented a work where the association between genetic polymorphisms and risk of dental caries is demonstrated for most of the salivary proteins. In addition, diabetic and hypertensive patients have dietary risk factors that can be caused by oral health problems. This affirmation was demonstrated in the work of Asif Ahmed et al. [10] through a statistical analysis using clinical data, in order to identify that patients with oro-dental problems were hemodynamically stressed.

In the present study, it is intended to analyze the determinants that affect this oral health situation based on dietary and demographic factors. Based on this general description of the oral health problem, specifically caries, the main contribution of this work is to determine that, given the socioeconomic and dietary features, using an ANN can determine if the patient presents an optimal state of health or with the presence of repairs or caries, obtaining a classification system that allows for knowing the differences in the features that affect the population, as well as looking for the future prediction of oral health.

This method is an important tool for human resources and dental services because it reflects information collected in the population groups studied, which could unveil that oral health problems are not simple conditions, but they are obtained from different processes that have their trigger, which can enhance or mitigate them, impacting positively or negatively on the state of oral and general health.

### Related Work

The condition of dental caries has been described in the scientific literature under different terms, as a multifactorial disease that is characterized by localized and progressive demineralization of the inorganic portions of the tooth and the subsequent deterioration of its organic part, with a high degree of morbidity and high prevalence [11,12,13].

A related work that suggests CADx to improve oral health prediction is developed by Zhang et al. [14], where an autoregressive integrated moving average model is performed and a grey predictive model for the estimation of the national prevalence of early childhood caries from 2014 to 2018, finding that the highest annual prevalence would be of 55.8%, being attributed to the socioeconomic developments and the public health service. In the work of Asif et al. [15], a caries preventive tool based on the influence of genetic pattern using the frequency of occurrence of fingerprint patterns among children, using as a validation measure the *p*-value, finding that dermatoglyphics could be an appropriate method to explore the possibility of a noninvasive and early predictor for dental caries. Chapple et al. [16] performed a systematic appraisal to identify potential risk factors for caries and periodontal diseases using genetic, role of diet and nutrition risk factors, obtained as results that the genetic contribution to these conditions present an attributable risk up to 50%, while controlled diabetes and obesity are common acquired factors. Hayes et al. [17] had as an objective to determine the risk indicators associated with root caries conducting a prospective longitudinal study using a regression analysis with data related to hygiene habits, diet, smoking habits and education level and as the outcome the root caries experience, suggesting a correlation between root caries and the variables poor plaque control, xerostomia, coronal decay and exposed root surfaces, obtaining a prevention tool for the root caries condition. In the work of Sudhir et al. [18], evaluating Caries Management by Risk Assessment (CAMBRA) as a tool for caries risk prediction was proposed, using as validation parameter OR value and ROC curve, obtaining that CAMBRA was found to be 47.62% with a specificity of 80% and AUC was found to be 0.638, which means that it is valid and highly predictive in determining the caries risk. Finally, Twetman [19] proposed to summarise the findings of recent systematic reviews covering caries risk assessment, finding that caries risk assessment should be carried out at the subject’s first dental visit and reassessments should be done during childhood; multivariate models display a better accuracy than the use of single predictors; there is no clearly superior method to predict future caries and no evidence to support the use of one model, program, or technology before the other; and the risk category should be linked to appropriate preventive care with recall intervals based on the individual need.

This work is organized as follows: Section 2 briefly describes the materials and methods used for the development of the data analysis, including data preprocessing, data classification and data evaluation. Section 3 exposes the results obtained. Finally, results are discussed in Section 4 and concluded in Section 5.

## 2. Materials and Methods

In this section, the process that was carried on for the classification between subjects with presence of caries and subjects with absence was presented, based on the information obtained from a series of socioeconomic and nutritional features. The databases that were used in this work were obtained from the National Health and Nutrition Examination Survey (NHANES, 2013–2014). In addition, the description of the subjects and the methods used for their classification are presented.

**Figure 1:**
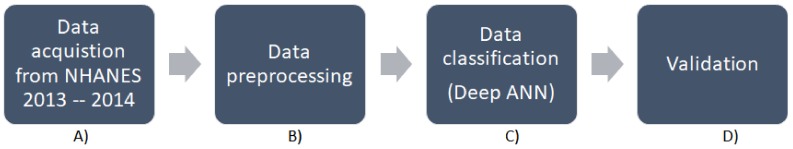
Flowchart of the methodology followed.

In Figure 1, the flowchart of the steps followed for the experimentation of this work is presented. Section (A) presents the data acquisition of the public databases “Demographic” and “Dietary” from NHANES 2013–2014. Section (B) represents the data preprocessing, where a feature reduction is performed due to the high amount of missing data and singular values that are presented, in addition to separating the data in two sets, one for training and one for testing. Section (C) refers to the data classification of subjects according to their oral health status. Finally, in Section (D), the evaluation of the ANN performance is presented, in order to know the accuracy with which the model classifies the subjects.

### 2.1. Features Description

NHANES is a program of studies designed to evaluate the health and nutritional status of children and adults in United States, and it was founded by Centers of Disease Control and Prevention (CDC) and National Center for Health Statistics (NCHS). The surveys conducted by this program are unique, since it combines interviews and physical exams [20].

NHANES collects information from different types of data, and, in turn, this information is included in six main contexts; demographic, dietetic, examination, laboratory, questionnaire and limited access. These contexts are contained by the information described as follows:

- Demographic: it provides individual, family and household level information in different topics (income of households and families, size of households and families, pregnancy status, among others).
- Dietetic: it provides detailed information on dietary intake, in order to estimate the types and amount of food and beverages consumed, in addition to estimating the intake of energy, nutrients, and other food components.
- Examination: it provides information of the health status, indicators of disease risk, and access to preventive and treatment services, from different aspects, including oral health.
- Laboratory: it provides information of the results obtained from laboratory analysis of different components (components of urine, proteins, triglycerides, plasma, among others).
- Questionnaire: it provides information of the data obtained from the interviews conducted through a system of computer-aided personal interview, of different topics (alcohol use, cardiovascular health, dermatology, among others).
- Limited access: it provides similar information to that found in the questionnaire; however, it isn’t publicly available.

There were 189 features analyzed for this work, of which 188 belonged to demographic data, described in Appendix A, and dietary data, described in Appendix B. These features were used as input variables for the classification of subjects, while the remaining feature belonged to examination data, describing the oral health status of the subjects based on the presence/restoration or absence of caries, for which it was used as an output feature.

### 2.2. Subjects Description

The subjects of these databases were submitted to a series of different questionnaires, related to the different features. These subjects belong to different counties in the USA and they were randomly selected with a computer algorithm by NHANES.

**Figure 2:**
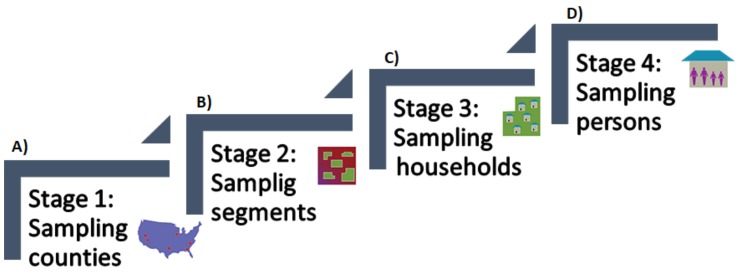
Flowchart of the stages followed by NHANES 2013–2014 for the sampling of participants.

Figure 2 presents a flowchart of the randomly selection process followed by NHANES. (A) presents the first stage that corresponds to the sampling of counties. In this step, all the counties are divided into 15 groups according to their characteristics. Then, from each group, one county is selected, forming the 15 counties in the NHANES surveys for each year. (B) presents the second stage, corresponding to the sampling of segments, where from each county smaller groups are formed (with a large number of households in each group), selecting between 20 and 24 of these groups. (C) presented the third stage, which is referred to the sampling of households, where from all of the houses that correspond to the groups that were selected in the past stage, a sample of about 30 households are selected within each group. Finally, (D) presents the fourth stage, corresponding to the sampling of persons, where the NHANES interviewers go to the selected households and ask for the information of the surveys (age, race, and gender). The members of the households that responded to the surveys were randomly selected through a computer algorithm; they can be selected some, all, or none of the household members.

The main target population for NHANES is the non-institutionalized civilian resident population of the USA. The design of the population selection is based on the sampling of a larger number of specific subgroups that present particular public health characteristics, in order to increase the reliability and precision of health status indicators. NHANES started these design changes in 2011, including in its population the oversampled subgroups survey cycle:

- Hispanic persons;
- Non-Hispanic black persons;
- Non-Hispanic Asian persons;
- Non-Hispanic white and other* persons at or below 130% of the poverty level; and
- Non-Hispanic white and other* persons aged 80 years and older.

This random selection of subjects avoid presenting any bias problem, favoring the important variety of demography characteristics of the subjects reducing the probability that the methodology followed could be influenced by the data used.

**Figure 3:**
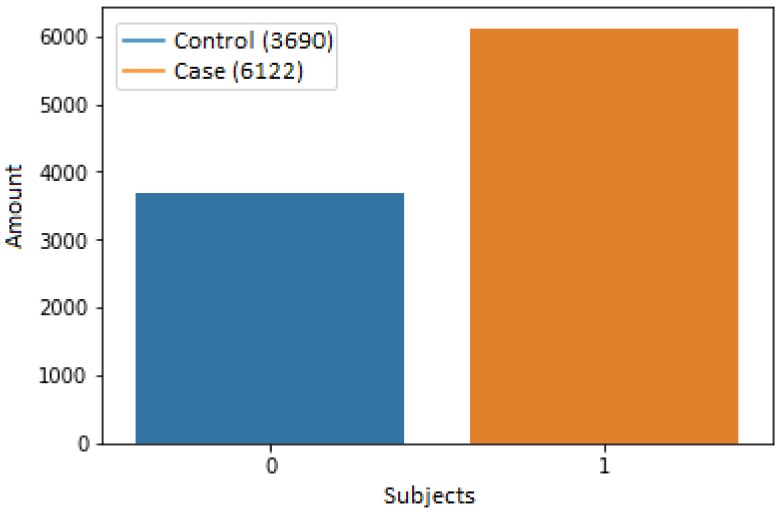
Graph of the total number of subjects used, classified according to their oral health status. The subjects contained in the bar with ’0’ presented absence of caries, while the subjects contained in the bar with ’1’ presented presence/restoration of caries.

Figure 3 presents the total of subjects contained in the database used. From 9812 subjects, 3690 were control subjects and 6122 were case subjects. The subjects were in an age range of 0 to 80 years old; 4830 belonged to the masculine gender and 4982 to the feminine gender.

It is important to mention that this specific database was chosen in order to show the consistency of the data and the results obtained in the relationship to its background work. In this work [21] three different models obtained through a fast backward selection (FBS) of features are presented, each model corresponding to a different age group, then a posterior evaluation using a net reclassification improvement (NRI) technique is performed, besides the AUC parameter, obtaining a maximum true positive-true negative rate of 0.787. On the other hand, this work [22] presents a multivariate model obtained through a FBS method based on the *p*-value, in order to classify between three different classes, ”caries”, ”restorations” and ”control”; this model was evaluated using a statistical analysis, obtaining a maximum AUC value of 0.664. Finally, this work [23] presents a univariate analysis using a linear regression approach in order to classify between subjects with the presence of caries and with absence; then, from the most significant univariate models, a multivariate model contained by three features was developed, which obtained an AUC value of 0.572.

### 2.3. Data Analysis

The data analysis of this work was performed through a multivariate approach, subjecting the demographic and dietary features to a deep ANN for the classification of subjects according to their health and then a statistical validation was carried out in order to evaluate the results obtained from the ANN developed for these specific data.

#### 2.3.1. Data Preprocessing

The data preprocessing consisted initially in the manual elimination of the features that presented a high percentage of missing values (>30%), represented as “Not a Number” (NaN). Then, of the remaining features, those that presented a low percentage of missing data (≤30%) were imputed with the ”rfimput” function, of the ”randomForest” package (version 4.6-12, 6 October 2015) [24], for R, which consists of replacing all NaN with the average of the values that present the column where the missing data is located.

The columns containing singular values were also removed because they didn’t provide significant information. Two values are singular if they have multiple values between them or if they present the same value in the whole column.

Finally, the database was separated into two sets, randomly and balanced selecting a set for the training stage, contained with 70% of the data and a set for the testing stage, contained with the remaining 30% of data.

#### 2.3.2. Data Classification

The classification of subjects was performed according to the presence or absence of caries through a dense ANN that was designed based on the data of the subjects, using the package ”Keras”. ”Keras” is a high-level ANN application programming interface written in Python. It was developed with the approach of allowing fast experimentation, facilitating the creation of prototypes in a simple way [25].

ANNs seek a solution to a specific task, based on the correlation between features, through learning or training that resembles the behavior of biological neural networks. Using different layers composed of nodes or neurons, the ANNs look for a model of the relationship between the input features and the output feature. The number of nodes that exist for each layer is configurable, as is the number of layers and, depending on the data, there is usually a greater capacity of the network, the greater the number of layers and neurons; however, if there isn’t the right amount of these elements, problems of overfitting can be caused .

ANNs present three main elements: (1) a set of synapses or connections, characterized by a ”weight”, where the input signal is connected to a neuron through its product with the weight of that connection; (2) an adder, which adds the contributions of a signal weighted by all the weights; and (3) an activation function, which is equivalent to a transfer function, affecting the neurons, allowing for limiting the amplitude of the network output, providing a permissible range for the output signal in terms of finite values. Among the most common activation functions are the lineal, quadratic, geometric, logistic, and rectified linear unit function, among others.

On the other hand, an ANN is dense when each node of a layer is connected with every node of the next layer; while the recurrence term refers to the network presenting at least one feedback loop, which will provide a deeper impact on the learning capacity of the network, as well as on its performance, since it allows for optimizing its behavior through a parameter that gives knowledge of the behavior of the data and allows for guiding the adjustment of the configuration of the network to improve its accuracy.

**Figure 4:**
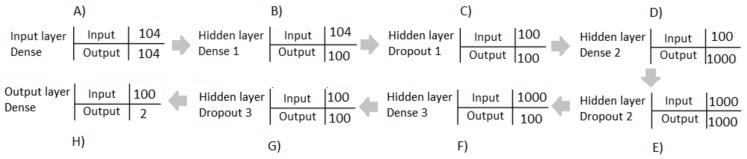
Diagram of the ANN designed.

The ANN designed was composed by a series of dense and dropout hidden layers, as shown in the diagram of Figure 4. The input layer (A) was assigned 104 neurons, making reference to the 104 features of the dataset. The first dense hidden layer (B) was composed of 100 neurons, as was the first dropout hidden layer (C), which had a loss percentage of 50%. The second dense hidden layer (D) was assigned 1000 neurons, as was the second dropout hidden layer (E), which had a loss percentage of 25%. The third dense hidden layer (F) was composed of 100 neurons, as was the third dropout hidden layer (G), which had a loss of 50%. Finally, the fourth dense hidden layer was the output layer (H), which was characterized by two neurons, making reference to the two outputs or possible classifications.

The input layer and the dense hidden layers used as activation function, the Rectified Linear Unit (ReLU) function, which consists of assigning 0 to the values of the neurons that are <0, and to respect the value of the neurons when their values are ≥0, as shown in Equation (1) [26]:

(1) $$\mathrm{ReLU}\left(x\right)=\left\{\begin{array}{cc} x, & \;x\geq 0,\\ 0, & \;x<0. \end{array}\right.$$

The output layer used the Normalized Exponential function as an activation function, also known as ”Softmax”, which represents a general form of the logistic function and it is used to compress a vector of arbitrary values into a vector of real values in a range of [0, 1]. This function is shown in Equation (2), where σ(z) represents the *K*-dimensional vector, *z*, of binary values [27]:

(2) $$\sigma {\left(z\right)}_{j}=\frac{e^{z_{j}}}{\sum_{k=1}^{K}e^{z_{k}}},\;j=1,\ldots ,K.$$

Finally, the dropout hidden layers were included in order to avoid overfitting problems, as mentioned before, and its performance is based on assigning the value of 0 to a percentage of the data and thus they are not taken into account for the classification of subjects in that layer. The data subset selected by these layers is changing with each iteration, causing that in each epoch the classification will be done omitting a different percentage of data. On the other hand, due to the recurrence of the ANN, it was possible to optimize its performance. The optimization algorithm, “Adam”, was selected, which bases its operation on stochastic gradient descent algorithms, making use of the average of the first and second moments of the gradients to adapt the rate of the learning parameter. Specifically, “Adam” calculates the exponential moving average of the gradient and the square gradient, and controls the decay rates of that moving average [28]. Some of the benefits that present “Adam” are that it is a method that is straightforward to implement, is computationally efficient, has little memory requirements, is invariant to diagonal rescaling of the gradients, and one of the most important points is that is suitable for problems that are large in terms of data or features. In addition, the parameters have intuitive interpretations and typically require little tuning [28].

It is important to mention that the number of iterations or epochs that the ANN will have for the optimization of its behavior, based on the recurrence, is also configurable and will depend on the type of data. To know what is the appropriate number of epochs, there are some parameters that allow for evaluating the behavior through each iteration, such as the loss function and the accuracy [29]. The number of the epochs that were selected through a series of tests with different numbers were 100 epochs, since they demonstrated having the best performance in terms of the accuracy of the ANN.

Based on the above, “keras” has the advantage that it allows for designing the architecture of the ANN according to the type of data, configuring the type of ANN, number of nodes, validation, loss function, among others [30,31].

#### 2.3.3. Evaluation

Finally, in order to validate the results of classification obtained by the ANN, three parameters were evaluated; loss function, accuracy and ROC curve. The loss function and accuracy were calculated on each epoch, allowing to know if the performance of the ANN was improving, while the ROC curve was calculated based on the average of the general performance of the ANN.

ANNs are mostly trained using gradient methods through an iterative process of decreasing the loss function. A loss is designed to have the main property that the lower its value, the better the model that fits its data and, in addition, it will be differentiable, which will optimize the network directly, giving information of the capacity of the system. Therefore, the loss function is based on the search of the global minimum, which corresponds to the minimum error, based on a learning factor. Some of the most common techniques to calculate the loss function are the mean square error, mean absolute error, binary cross-entropy, and Poisson, among others [31].

The loss function that was used is preset in “keras” as “binary-crossentropy” and calculates the cross-entropy value for binary classification problems. This method uses the Kullback–Leibler distance, which is a measure between two density functions *g* and *h*, known as the cross-entropy between *g* and *h*, as shown in Equation (3). Its operation is based on iterations, generating a random set of values estimating the value that wants to be obtained and then actualizing the parameters in the next iteration to generate “better” values or more approximate, in terms of the Kullback–Leibler distance [32]:

(3) $$D\left(g,h\right)=\int g\left(x\right)ln\frac{g(x)}{h(x)}\mu \left(dx\right)=\int g\left(x\right)lng\left(x\right)\mu \left(dx\right)-\int g\left(x\right)lnh\left(x\right)\mu \left(dx\right).$$

On the other side, the accuracy allows for measuring the performance of the ANN through a non-differentiable function. This metric doesn’t allow to optimize the network, but it allows to select the model that shows the most suitable performance in the training of the network, and it is based on the calculation of the average of the differences that exist between the classification calculated by the ANN model and the true classification of the data, as shown in Equation (4), where the accuracy is reported as 1-error. $V_{pred}$ refers to the classification value that was calculated by the ANN, while $V_{actual}$ refers to true classification value [33]:

(4) $$error=V_{pred}-V_{true}.$$

In this work, the accuracy was calculated with the “binary-accuracy” function from “keras”, which calculates the average accuracy rate across all predictions for binary classification problems.

The ROC curve is a standard method that allows for evaluating the precision with which the model classifies, based on the relationship between sensitivity and specificity. Sensitivity is defined as the proportion of subjects with a condition that were classified as positive, which means the positive predictive values (PPV); this value is calculated with Equation (5), where TP represents the number of true positives and FP represents the number of false positives [34,35]:

(5) $$PPV=\frac{TP}{TP+FP}.$$

Specificity is defined as the proportion of subjects without a condition that was classified as negative; this means the negative predictive values (NPV); this value is calculated with Equation (6), where TN represents the number of true negatives and FN represents the number of false negatives [34,35]:

(6) $$NPV=\frac{TN}{TN+FN}.$$

The ROC curves were calculated to obtain the true positive and true negative rate for each class, for the macro-average precision and for the micro-average precision.

The macro-average precision is obtained through the sum of the true positives, false positives and false negatives of the system, for different sets, as shown in Equation (7), where $TP_{1}$ refers to the true positives of the set one, $TP_{2}$ refers to the true positives of the set two, $FP_{1}$ refers to the false positives of the set one and $FP_{2}$ refers to the false positives of the set two [36,37]:

(7) $$\text{Micro-average}=\frac{TP_{1}+TP_{2}}{TP_{1}+TP_{2}+FP_{1}+FP_{2}}.$$

On the other hand, the macro-average precision is a direct method; it takes the average of the accuracy of the system in different sets. It is calculated with Equation (8), where $A_{1}$ refers to the average of the set one and $A_{2}$ refers to the average of the set two [36,37]:

(8) $$\text{Micro-average}=\frac{A_{1}+A_{2}}{2}.$$

The analysis of this work was performed in Python (version 3.6), using the packages, “Keras” (version 2.1.5) [25], “Scipy” (version 1.0.0) [38], “Pandas” (version 0.22.0) [39] and “Sklearn” (version 0.19.1) [36].

## 3. Results

The two databases that were used in this work were contained by a total of 188 demographic and dietary features, besides a feature being used that was contained by the oral health status as an output feature for the classification of subjects.

After the data preprocessing, only 105 features were conserved, of which 25 belonged to demographic features and 79 to dietary features, the remaining feature refers to the output feature.

**Figure 5:**
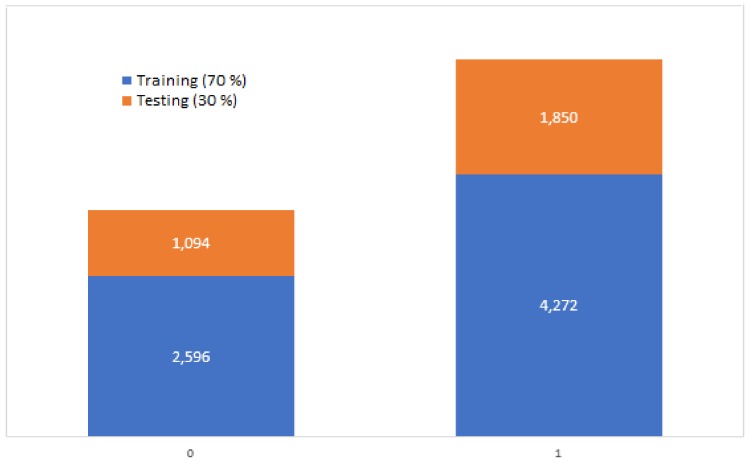
Graph of the number of subjects that belongs to each dataset, training and testing. The content in the bar with “0” presented a status of absence of caries, while the contest in the bar with “1” presented a status of presence/restoration of caries.

Of the total set of 9812 subjects, 3690 belonged to controls and 6122 belonged to cases. This dataset was divided into two subsets, one for training which was contained by 70% of the data (2596 controls/4272 cases), and one for testing which was contained with the remaining 30% of the data (1094 controls/1850 cases). These data are graphically shown in Figure 5.

The dataset that was designed for the training stage was evaluated at each of the 100 epochs through the accuracy and loss function values. This number of epochs was selected through a comparison between the values obtained using different number of epochs; Table 1 presents the values obtained for ten different epochs.

On the other hand, in Table 2, a comparison of results using different number of layers and neurons is shown. The number of epochs selected was 100, establishing this number according to the result obtained from the previous table. It is possible to observe that the most statically significant values of accuracy and loss function were obtained using an ANN designed with seven layers, four dense layers and three dropout layers. The dense layers were contained by 104, 1000, 100 and two neurons, in descendant order, while the dropout layers represented 0.50, 0.25 and 0.50 percentages, in descendant order too.

**Figure 6:**
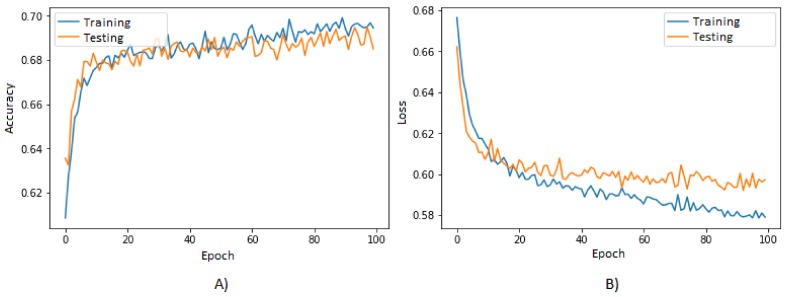
Graphs of the performance of the accuracy (**A**) and the loss function (**B**) of the ANN in each epoch.

The graph of the performance of the accuracy is shown in Figure 6A, where the blue line represents the behavior of the training data, obtaining a final accuracy of 0.69, while the orange line represents the behavior of the testing data, obtaining a final accuracy of 0.68.

The graph of the performance of the loss function is shown in Figure 6B, where the blue line represents the behavior of the training data, obtaining a final value of 0.58, while the orange line represents the behavior of the testing data, obtaining a final value of 0.60.

Both accuracy and loss function values were obtained in order to know the performance of the classification of subjects on each iteration; nevertheless, the accuracy is a parameter that may show an optimistic response if the data presents any bias. Therefore, there were also measured the specificity and sensitivity parameters in order to ensure that the classification of subjects was statistically significant.

**Figure 7:**
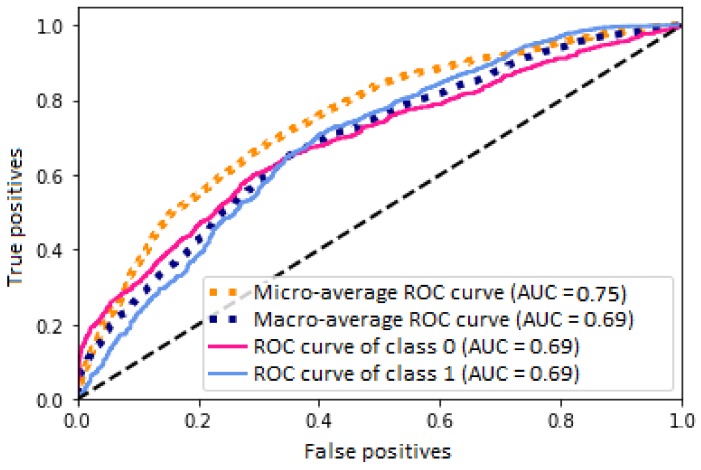
Graph of the ROC curves generated based on the average performance of the ANN in the classification of subjects.

The graph of the Figure 7 shows the ROC curves of the mean performance of the ANN, where the pink line refers to the proportion of sensitivity and specificity for the class “0”, which are the subjects that presented absence of caries, obtaining an AUC value of 0.69. The light blue line refers to the proportion of sensitivity and specificity for the class “1”, which are the subjects that presented presence/restoration of caries, obtaining an AUC value of 0.69. The dotted orange line refers to the curve calculated with the micro-average of the proportion of sensitivity and specificity for the classification of subjects, obtaining an AUC value of 0.75; the dotted dark blue line refers to the curve calculated with the macro-average, obtaining an AUC value of 0.69.

## 4. Discussion

Of the two datasets that were obtained through the preprocessing step, it is possible to observe that the set of cases contains more data than the set of controls as it is observed in Figure 5; this was due to the fact that the cases included the subjects with presence of caries and with restorations. Nevertheless, the number of subjects that were contained on each dataset was enough to train the ANN, obtaining statistically significant results.

The data were separated in two sets based on an aleatory and balanced selection; one of the sets was contained with 70% of the data and the other dataset was contained with 30%, as shown in Figure 5. The dataset that was contained with the largest amount of data had the purpose of training the ANN. The dense ANN was trained during 100 epochs and was optimized through the optimization algorithm, “Adam”, which had the purpose of improving the performance of the ANN with feedback.

Finally, for the validation of the ANN modeling, the accuracy and the loss function in each of the epochs with the dataset that was contained with the least amount of data were obtained. The loss function was calculated using cross-entropy in order to know how the performance of the ANN was modified. The validation was carried out in both datasets, training and testing, in order to ensure that there was no adjustment in the classification of the subjects; that is, if the performance of these parameters is similar for both datasets, it means that the ANN is classifying based on a generalized model, since it manages to classify with an accuracy and a loss function similar in the training dataset and in the testing dataset.

In the graph of Figure 6A, it is observed that the performance of the accuracy is similar for both datasets, being slightly higher for the training data, which is normal since they are the data on which the ANN modeling is being based. As the performance is similar for both datasets, it is possible to know that the ANN model became generalized through the learning by being able to classify unknown data with a similar accuracy to that obtained with the training data. The final value obtained for the accuracy was 0.69, which is statistically significant since it means that at least 69% of the data was correctly classified.

On the other hand, in the graph of Figure 6B, it is observed that the performance of the loss function is also similar for both datasets. It is notable that, for the training dataset, this parameter is smaller compared to that obtained with the testing dataset; however, as in the case of the accuracy parameter, this performance is normal because the testing dataset was unknown for the model. In addition, having a similar performance allows for knowing that the model doesn’t have overfitting problems and that it manages to classify unknown data in a similar way as it classifies known data. The final value obtained for the accuracy had an average of 0.59. This value isn’t close to the ideal, which is zero; however, it is possible to observe that the data remains tending toward zero for both datasets, showing that the testing dataset decreases slower than the training dataset. This problem can be solved increasing the amount of data, removing features that are not significant or that are redundant for the model and increasing the size if the ANN or the number of epochs.

Although the graph of the loss function doesn’t present very favorable results, it allows for knowing that the modeling of the ANN is robust among the two datasets, in addition to continuing to decrease the loss function and reducing the error through the epochs, which is one of the main purposes of the training stage.

Finally, Figure 7 shows that all curves presented statistically significant values, obtaining AUC values ≥0.69. These curves are generated based on the proportion of true positives and true negatives, where for class “0”, class “1” and the macro-average, an AUC value of 0.69 was obtained, which implies that 69% of the subjects were classified appropriately for each of the classes and for their general average, while. for the curve generated for the micro-average, an AUC value of 0.75 was obtained, which implies that 75% of the subjects were classified appropriately when the measure was based in subsets of data. The ROC curves of class “0” and class “1” presented a very similar performance, besides obtaining the same AUC value, which means that the classification performance is equitable and the generalization of the model allows for classifying subjects of both classes.

On the other hand, the AUC value is greater for the micro-average than for the macro-average; this may be because the calculation of the macro-average is based in obtaining the true positives/true negatives proportion through the whole amount of data, while, for the micro-average, the calculation of the ROC curve is based in subsets of data, where some subsets can present better AUC values than obtained with the whole set, thus achieving a better average of classification.

Is it important to remark that this generalized model presents an impartial performance, since it was trained with data that cover a reasonable spectrum of the possible outcomes; in addition, the moment of time and geographical location are parameters that were taken into account in the initial data acquisition; regardless of whether the data was acquired in the USA, most of the subjects were from other regions, of which a percentage belongs to Mexican subjects, providing robustness in the results and avoiding any bias problem related to the structure, the moment of time and the geographical location of the data.

Finally, the results obtained allow for supporting the main motivation of this work, which was to develop a tool for human resources and dental services, since this model is able to give a statistically significant response of the possible development of caries based on information that doesn’t require any extra equipment for its acquisition, only the willingness of people to answer the demographic and diet surveys, providing support to the dental specialists in the work of reducing the high incidence of dental caries

Based on these discussions, it can be proposed as future work to add a stage in the methodology that consists of a step for the feature selection using a genetic algorithm approach. This stage will help to remove redundant information and correlated features, keeping only those features that provide the most significant information for the classification of subjects, besides reducing the computational cost. Additionally, for the validation of the feature selection, a forward selection and backward elimination may be used in order to test the accuracy of the set of features obtained through the selection process, ensuring the certainty of the model behavior.

## 5. Conclusions

According to the results obtained, it is possible to conclude that the amount of data that was used to model the classification of the subjects was adequate for the preliminary generalized learning of the ANN, basing this on the performance that the classification of cases had, being similar to the classification of controls. This performance was observed in the accuracy and loss function graphs, which allows for recognizing that the number of epochs that were assigned to the ANN was sufficient to maintain stable performance of the parameters in both datasets.

The accuracy achieved with this modeling was around 0.70, a value that is statistically significant since it implies that 70% of the time will be correctly classified to the subjects with presence or absence/restoration of caries.

The loss function decreased by approximately 10% since the beginning of the training, showing minor changes throughout the epochs, implying an approximation to the global minimum sought.

As a final evaluation, all the ROC curves obtained an AUC value statistically significant, implying that, around 70% of the time, subjects were correctly classified, according to the true positives and true negatives proportion, a value that corroborates the accuracy obtained. Nevertheless, a methodology was proposed based in convolutional neural networks in order to identify features in a spectral or spatial domain to obtain time-independent abstract features, looking for the improvement in the modeling for the classification of subjects.

Based on this, it is possible to classify subjects with the absence of caries from subjects with presence/restorations, through demographic and dietary data, with a statistically significant accuracy, demonstrating that the socioeconomic and nutritional status are important determinants in the development of caries.

Then, according to the results obtained, it was demonstrated that the demographic situation can significantly affect the prevalence of dental caries. Based on this, the analysis of oral health data from exclusively Mexican subjects is proposed, comparing those results with the ones obtained in this work, with the purpose of proving how demography can influence the oral health status.

On the other hand, even though the results do not show an ideal behavior, they give preliminary knowledge of the benefit that its implementation would have in a real environment, since the requirements for its use are minimal, no in-depth knowledge of the techniques used is required, results are presented quickly and the computational cost is very low, besides the accuracy obtained being statistically significant. In addition, an important point to take into account is that the performed experimental tests were based in a real production environment, since all the subjects that were part for the development of this work were real control and cases, and the demographic and dietary databases were obtained from real information.

The hardware tool that is required for the implementation of this work is a computer, while the free software tool that is required is Python, which is a programming language that allows for working quickly and integrating systems more effectively. As extra information, it is mentioned that the computer that was used for the development of this work was a laptop Acer Aspire F5-573-70LX 15.6” (Acer America Corporation, 333 West San Carlos Street, Suite 1500, San Jose, CA 95110), Intel Core i7-7500U 2.70 GHz (Plot 6, Bayan Lepas Technoplex Medan Bayan Lepas 11900 Bayan Lepas Penang, Malaysia, Georgetown, Pulau Pinang, Malasia), 16 GB, 1 TB + 128 GB Solid State Drive, Windows 10 Home (15010 NE 36th Street, Microsoft Campus, Building 92, Redmond, WA 98052), 64-bit; and the version of Python that was used is 3.6 [40].

Finally, based on the last point, it is evident that the implementation of the model proposed in this work could provide an easy, free and fast tool that helps the specialists in the preventive diagnosis of dental caries, besides offering an option that collaborates in the decrement of the incidence of this public health problem.

## Figures and Tables

**Table 1:** Comparison of the accuracy and loss function values obtained using different number of epochs.

Epochs	Accuracy	Loss Function	Processing Time (s)
10	0.67	0.60	10.44
30	0.68	0.60	32.02
50	0.68	0.59	55.46
80	0.69	0.59	93.57
100	0.69	0.58	124.55
150	0.68	0.60	188.21
200	0.68	0.61	246.74
300	0.69	0.62	369.06
500	0.70	0.62	646.86
1000	0.70	0.66	4152.96

**Table 2:** Comparison of the accuracy and loss function values obtained using different number of layers and neurons.

Layers Dense/Dropout	Neurons	Accuracy	Loss Function	Processing Time (s)
2/1	104>0.50>2	0.68	0.60	36.84
3/1	104>0.50>1000>2	0.68	0.59	71.10
3/2	104>0.25>1000>0.50>2	0.69	0.61	93.86
4/1	104>1000>0.50>100>2	0.68	0.73	117.63
4/2	104>0.50>1000>0.50>100>2	0.68	0.59	119.77
4/3	104>0.50>1000>0.25>100>0.50/2	0.69	0.58	124.55
5/1	104>100>1000>0.50>100>2	0.68	0.91	129.53
5/2	104>100>0.50>1000>0.25>100>2	0.68	0.65	131.66
5/3	104>100>0.50>1000>0.25>100>0.50>2	0.69	0.64	139.37
5/4	104>0.25>100>0.50>1000>0.25>100>0.50>2	0.68	0.59	138.38

## Appendix A

**Table A1:** Description of demographic features.

Feature	Description
AIALANGA	Language of the MEC ACASI Interview Instrument.
DMDBORN4	In what country was Sample Person (SP) born?
DMDCITZN	Is SP a citizen of the United States?
DMDHHSIZ	Total number of people in the Household.
DMDHHSZE	Number of adults aged 60 years or older in the household.
DMDEDUC2	Highest grade or level of school completed or the highest degree received.
DMDHHSZB	Number of children aged 6–17 years old in the household (HH).
DMDHSEDU	HH reference person’s spouse’s education level.
DMDMARTL	Marital status.
DMDFMSIZ	Total number of people in the Family.
DMDHHSZA	Number of children aged 5 years or younger in the household.
DMDHRGND	HH reference person’s gender.
DMDHREDU	HH reference person’s education level.
DMDEDUC3	Highest grade or level of school completed or the highest degree received.
DMDHRAGE	HH reference person’s age in years.
DMDHRBR4	HH reference person’s country of birth.
DMDHREDU	HH reference person’s education level.
DMDHRMAR	HH reference person’s marital status.
DMQADFC	Did SP ever serve in a foreign country during a time of armed conflict or on a humanitarian or peace-keeping mission?
DMDYRSUS	Length of time the participant has been in the US.
DMQMILIZ	Has SP ever served on active duty in the U.S. Armed Forces, military Reserves, or National Guard?
FIAINTRP	Was an interpreter used to conduct the Family interview?
FIALANG	Language of the Family Interview Instrument.
FIAPROXY	Was a Proxy respondent used in conducting the Family Interview?
INDFMPIR	A ratio of family income to poverty guidelines.
INDHHIN2	Total household income (reported as a range value in dollars).
INDFMIN2	Total family income (reported as a range value in dollars).
LATVPSU	Variance unit: PSU variable for variance estimation.
LATVSTRA	Variance unit: stratum variable for variance estimation.
MIAINTRP	Was an interpreter used to conduct the MEC CAPI interview?
MIALANG	Language of the MEC CAPI Interview Instrument.
MIAPROXY	Was a Proxy respondent used in conducting the MEC CAPI Interview?
RIAGENDR	Gender of the participant.
RIDAGEEX	Age in years of the participant at the time of examination. Individuals aged 959 months and older are topcoded at 959 months.
RIDAGEMN	ge in months of the participant at the time of screening. Reported for persons aged 24 months or younger at the time of exam.
RIDRETH3	Recode of reported race and Hispanic origin information, with Non-Hispanic Asian Category.
RIDEXMON	Six month time period when the examination was performed.
RIDEXPRG	Pregnancy status for females between 20 and 44 years of age at the time of MEC exam.
RIDAGEYR	Age in years of the participant at the time of screening.
RIDRETH1	Recode of reported race and Hispanic origin information.
RIDEXAGM	Age in months of the participant at the time of examination.
RIDSTATR	Interview and examination status of the participant.
SDMVPSU	Masked variance unit pseudo-PSU variable for variance estimation.
SDDSRVYR	Data release cycle.
SDMVSTRA	Masked variance unit pseudo-stratum variable for variance estimation.
WTLAF8YR	Subsample 8-year fasting weight for participants aged 12 years and older who were examined in the morning sessions in Los Angeles, CA, USA.
WTLAI8YR	Full sample 8-year interview weight for participants in Los Angeles, CA, USA.
WTLAM8YR	Full sample 8-year MEC exam weight for participants in Los Angeles, CA, USA.

## Appendix B

**Table A2:** Description of dietary features.

Feature	Description
WTDRD1	Dietary day one sample weight.
DBQ095Z	Type of salt usually add to food at the table.
DR1TKCAL	Energy (kcal).
DR1TSUGR	Total sugars (g).
DR1TFIBE	Dietary fiber (g).
DR1TSFAT	Total saturated fatty acids (g).
DR1TMFAT	Total monounsaturated fatty acids (g).
DR1TPFAT	Total polyunsaturated fatty acids (g).
DR1TLYCO	Lycopene (μg).
DR1TFA	Folic acid (μg).
DR1TB12A	Added vitamin B12 (μg).
DR1_300	Was the amount of food that you ate yesterday much more than usual, usual, or much less than usual?
DR1_320Z	Total plain water drank yesterday—including plain tap water, water from a drinking fountain, water from a water cooler, bottled water, and spring water.
DR1_330Z	Total tap water drank yesterday—including filtered tap water and water from a drinking fountain.
DR1BWATZ	Total bottled water drank yesterday (g).
DR1DAY	Intake day of the week.
DR1DBIH	Number of days between intake day and the day of family questionnaire administered in the household.
DR1SKY	What type of salt was it? (Was it ordinary or seasoned salt, lite salt, or a salt substitute?).
DR1STY	Did SP add any salt to her/his food at the table yesterday?
DR1TACAR	Alpha-carotene (μg).
DR1TBCAR	Beta-carotene (μg).
DR1TCAFF	Caffeine (mg).
DR1TCALC	Calcium (mg).
DR1TCOPP	Copper (mg).
DR1TCRYP	Beta-cryptoxanthin (μg).
DR1TFDFE	Folate as dietary folate equivalents (μg).
DR1TFF	Food folate (μg).
DR1TFOLA	Total folate (μg).
DR1TLZ	Lutein + zeaxanthin (μg).
DR1TM161	MFA 16:1 (Hexadecenoic) (g).
DR1TM181	MFA 18:1 (Octadecenoic) (g).
DR1TM201	MFA 20:1 (Eicosenoic) (g).
DR1TM221	MFA 22:1 (Docosenoic) (g).
DR1TMAGN	Magnesium (mg).
DR1TMOIS	Moisture (g).
DR1TNIAC	Niacin (mg).
DR1TNUMF	Total number of foods/beverages reported in the individual foods file.
DR1TP182	PFA 18:2 (Octadecadienoic) (g).
DR1TP183	PFA 18:3 (Octadecatrienoic) (g).
DR1TP184	PFA 18:4 (Octadecatetraenoic) (g).
DR1TP204	PFA 20:4 (Eicosatetraenoic) (g).
DR1TP205	PFA 20:5 (Eicosapentaenoic) (g).
DR1TP225	PFA 22:5 (Docosapentaenoic) (g).
DR1TP226	PFA 22:6 (Docosahexaenoic) (g).
DR1TPOTA	Potassium (mg).
DR1TRET	Retinol (μg).
DR1TS040	SFA 4:0 (Butanoic) (g).
DR1TS060	SFA 6:0 (Hexanoic) (g).
DR1TS100	SFA 10:0 (Decanoic) (g).
DR1TS120	SFA 12:0 (Dodecanoic) (g).
DR1TS140	SFA 14:0 (Tetradecanoic) (g).
DR1TS180	SFA 18:0 (Octadecanoic) (g).
DR1TSELE	Selenium (μg).
DR1TSODI	Sodium (mg).
DR1TTHEO	Theobromine (mg).
DR1TVARA	Vitamin A as retinol activity equivalents (μg).
DR1TVB1	Thiamin (Vitamin B1) (mg).
DR1TVB12	Vitamin B12 (μg).
DR1TVB2	Riboflavin (Vitamin B2) (mg).
DR1TVB6	Vitamin B6 (mg).
DR1TVC	Vitamin C (mg).
DR1TVK	Vitamin K (μg).

**Table A3:** Description of dietary features.

Feature	Description
DR1TWS	When you drink tap water, what is the main source of the tap water? Is the city water supply; a well or rain cistern; a spring; or something else?
DR1TZINC	Zinc (mg).
DRABF	Indicates whether the sample person was an infant who was breast-fed on either of the two recall days.
DRD340	During the past 30 days did you eat any types of shellfish listed on this card?
DRD350A	Clams eaten during the past 30 days.
DRD350AQ	Number of times clams were eaten in the past 30 days.
DRD350B	Crabs eaten during the past 30 days.
DRD350BQ	Number of times crab was eaten in the past 30 days.
DRD350C	Crayfish eaten during the past 30 days.
DRD350CQ	Number of times crayfish was eaten in the past 30 days.
DRD350D	Lobsters eaten during the past 30 days.
DRD350DQ	Number of times lobster was eaten in the past 30 days.
DRD350E	Mussels eaten during the past 30 days.
DRD350EQ	Number of times mussels were eaten in the past 30 days.
DRD350F	Oysters eaten during the past 30 days.
DRD350FQ	Number of times oysters were eaten in the past 30 days.
DRD350G	Scallops eaten during the past 30 days.
DRD350GQ	Number of times scallops were eaten in the past 30 days.
DRD350H	Shrimp eaten during the past 30 days.
DRD350HQ	Number of times shrimp was eaten in the last 30 days.
DRD350I	Other shellfish ( ex. octopus, squid) eaten during the past 30 days.
DRD350IQ	Number of times other shellfish (ex. octopus, squid) was eaten in the past 30 days.
DRD350J	Other unknown shellfish eaten during the past 30 days.
DRD350JQ	Number of times other unknown shellfish was eaten in the past 30 days.
DRD350K	Refused to give detailed information on shellfish eaten during the past 30 days.
DRD360	During the past 30 days did you eat any types of fish listed on this card?
DRD370A	Breaded fish products eaten during the past 30 days.
DRD370AQ	Number of times breaded fish products were eaten in the past 30 days.
DRD370B	Tuna eaten during the past 30 days.
DRD370BQ	Number of times tuna was eaten in the past 30 days.
DRD370C	Bass eaten during the past 30 days.
DRD370CQ	Number of times bass was eaten in the past 30 days.
DRD370D	Catfish eaten during the past 30 days.
DRD370DQ	Number of times catfish was eaten in the past 30 days.
DRD370E	Cod eaten during the past 30 days.
DRD370EQ	Number of times cod was eaten in the past 30 days.
DRD370F	Flatfish eaten during the past 30 days.
DRD370FQ	Number of times flatfish was eaten in the past 30 days.
DRD370G	Haddock eaten during the past 30 days.
DRD370GQ	Number of times haddock was eaten in the past 30 days.
DRD370H	Mackerel eaten during the past 30 days.
DRD370HQ	Number of times mackerel was eaten in the past 30 days.
DRD370I	Perch eaten during the past 30 days.
DRD370IQ	Number of times perch was eaten in the past 30 days.
DRD370J	Pike eaten during the past 30 days.
DRD370JQ	Number of times pike was eaten in the past 30 days.
DRD370K	Pollock eaten during the past 30 days.
DRD370KQ	Number of times pollock was eaten in the past 30 days.
DRD370TQ	Number of times other type of fish was eaten in the past 30 days.
DRD370V	Refused to give detailed information on fish eaten during the past 30 days.
DRQSDIET	Are you currently on any kind of diet, either to lose weight or for some other health-related reason?
DRQSDT1	What kind of diet are you on? (Is it a weight loss or a low calorie diet: low fat or cholesterol diet; low salt or sodium diet; sugar free or low sugar diet; low fiber diet; high fiber diet; diabetic diet; or another type of diet?).
DRD370L	Porgy eaten during the past 30 days.
DRD370LQ	Number of times porgy was eaten in the past 30 days.
DRD370M	Salmon eaten during the past 30 days.
DRD370MQ	Number of times salmon was eaten in the past 30 days.
DRD370N	Sardines eaten during the past 30 days.
DRD370NQ	Number of times sardines were eaten in the past 30 days.
DRD370O	Sea bass eaten during the past 30 days.
DRD370OQ	Number of times sea bass was eaten in the past 30 days.
DRD370U	Other unknown type eaten during the past 30 days.

**Table A4:** Description of dietary features.

Feature	Description
WTDR2D	Dietary two-day sample weight.
DR1DRSTZ	Dietary recall status.
DR1TPROT	Protein (g).
DR1TCARB	Carbohydrate (g).
DR1TTFAT	Total fat (g).
DR1TCHOL	Cholesterol (mg).
DR1TFA	Folic acid (μg).
DR1TCHL	Total choline (mg).
DR1TIRON	Iron (mg).
DBD100	Frequency with which ordinary salt is added to the food on the table.
DRQSPREP	Frequency with which ordinary salt or seasoned salt is added in cooking or preparing foods in the household.
DR1TALCO	Alcohol (g).
DR1TS080	SFA 8:0 (Octanoic) (g).
DR1TATOC	Vitamin E as alpha-tocopherol (mg).
DR1TATOA	Added alpha-tocopherol (Vitamin E) (mg).
DR1TVD	Vitamin D (D2 + D3) (μg).
DR1TPHOS	Phosphorus (mg).
DR1TS160	SFA 16:0 (Hexadecanoic) (g).
DRD370UQ	Number of times other unknown type of fish was eaten in the past 30 days.
DRD370V	Refused to give detailed information on fish eaten during past 30 days.
DRDINT	Indicates whether the sample person has intake data for one or two days.
DRQSDIET	Are you currently on any kind of diet, either to lose weight or for some other health-related reason?
